# Pneumolysin Attenuates Lethal *Streptococcus pneumoniae* Superinfection Following SARS-CoV-2 Infection

**DOI:** 10.3390/microorganisms14102131

**Published:** 2026-09-22

**Authors:** Austin W. Todd, Ekaterina Koroleva, Danielle B. Molotsky, Brianna N. Faz, Anna A. Korchagina, Sergey A. Shein, Yan Xiang, Thirumalai R. Kannan, Alexei V. Tumanov

**Affiliations:** Department of Microbiology, Immunology, and Molecular Genetics, University of Texas Health Science Center at San Antonio (UTHSCSA), San Antonio, TX 78229, USA; todda1@livemail.uthscsa.edu (A.W.T.); koroleva@uthscsa.edu (E.K.); molotsky@livemail.uthscsa.edu (D.B.M.); brianna.faz@utsouthwestern.edu (B.N.F.); korchagina@uthscsa.edu (A.A.K.); shein@uthscsa.edu (S.A.S.); xiangy@uthscsa.edu (Y.X.); kannan@uthscsa.edu (T.R.K.)

**Keywords:** co-infection, superinfection, SARS-CoV-2, *Streptococcus pneumoniae*, pneumolysin, lung pathology

## Abstract

*Streptococcus pneumoniae* (*Spn*) is a common bacterial co-infection in patients with respiratory viral infections, including influenza and SARS-CoV-2, and is associated with increased morbidity and mortality. However, the mechanisms underlying the severe disease caused by SARS-CoV-2 and *Spn* co-infection remain poorly understood. Here, we show that *Spn* infection 3 days after infection with mouse-adapted SARS-CoV-2 MA10 markedly increased morbidity and mortality in wild-type mice. In contrast, SARS-CoV-2 infection 3 days after *Spn* infection did not increase mortality, demonstrating that disease severity depends on the sequence of infection. Increased susceptibility to *Spn* following SARS-CoV-2 infection was associated with enhanced viral burden, exacerbated lung pathology, pronounced neutrophil accumulation, and increased expression of IFN-β and the neutrophil-recruiting chemokine CXCL2. To determine whether pneumolysin (PLY), a major *Spn* virulence factor, contributes to superinfection-induced pathology, we infected mice with a PLY-deficient *Spn* strain. Unexpectedly, PLY deficiency further increased mortality and lung pathology without altering bacterial or viral titers. Immunofluorescence analysis revealed increased accumulation of neutrophils and macrophages in the lungs of mice co-infected with PLY-deficient *Spn*. These results indicate that *Spn* superinfection exacerbates SARS-CoV-2-induced lung disease, whereas PLY unexpectedly attenuates disease severity. We propose that PLY limits immunopathology during SARS-CoV-2–*Spn* superinfection by restraining macrophage- and neutrophil-mediated inflammation.

## 1. Introduction

The overall prevalence of bacterial co-infection at hospital admission for SARS-CoV-2 is relatively low, estimated at about 5% in major global meta-analyses [1,2]. However, secondary hospital-acquired bacterial superinfections occur in 15–20% of patients during longer inpatient stays, particularly among critically ill patients [3], and can affect 20–40% of patients in intensive care units [4]. COVID-19 patients with bacterial co-infections or secondary infections have higher in-hospital mortality and a longer length of stay [5,6]. The most frequently isolated organisms include *Streptococcus pneumoniae* (*Spn*), *Staphylococcus aureus*, *Pseudomonas aeruginosa*, and *Klebsiella pneumoniae* [2,3,4]. Timely and appropriate antibiotic use, supported by accurate diagnosis, is crucial for improving patient outcomes and preventing antimicrobial resistance [5]. Antimicrobial resistance is also highly prevalent in patients with COVID-19 and poses a significant treatment challenge [7]. Additionally, viral co-infections, such as influenza, can exacerbate disease in COVID-19 patients [8] and in animal co-infection models [9]. Although the synergistic effects of co-infections on SARS-CoV-2 disease severity are well established, the cellular and molecular mechanisms remain incompletely understood.

Accumulating clinical evidence suggests that *Spn* exacerbates SARS-CoV-2-induced lung pathology and contributes to disease severity [1,6,10,11,12,13,14,15,16,17]. Studies of SARS-CoV-2 and *Spn* co-infection in K18-hACE2 transgenic mice showed increased mortality, indicating synergistic effects [18,19]. However, the mechanisms by which *Spn* infection promotes SARS-CoV-2-induced mortality and lung disease remain unclear. Furthermore, the impact of *Spn* co-infection on the pathogenesis of a mouse-adapted SARS-CoV-2 strain in immunocompetent wild-type (WT) mice remains unknown.

Pneumolysin (PLY), a cholesterol-dependent pore-forming toxin produced by *S. pneumoniae*, is a major determinant of pneumococcal virulence and has been implicated in epithelial injury, inflammatory activation, and tissue damage during bacterial pneumonia [20,21,22,23]. High PLY concentrations directly disrupt host cell plasma membranes and mitochondria; trigger inflammasome activation; and promote the production of proinflammatory cytokines and chemokines, including IL-1β, IL-18, TNF, CXCL2, CCL5, and CCL8 [23,24,25,26,27]. However, PLY can also promote anti-inflammatory responses by binding to mannose receptor C type 1 (MRC-1) on macrophages and dendritic cells [28], triggering TGF-β production by epithelial cells [29] and activating autophagy [30]. The dual role of PLY during pneumococcal infection is well established [23,31]. Despite extensive knowledge of PLY biology in pneumococcal infection and influenza-associated secondary bacterial pneumonia, its specific contribution to SARS-CoV-2/*Spn* superinfection remains undefined.

To investigate the impact of *Spn* co-infection on SARS-CoV-2 pathogenesis in WT mice, we used the mouse-adapted MA10 strain of SARS-CoV-2 [32] and administered *Spn* either 3 days before or 3 days after SARS-CoV-2 infection. Our results show that the timing of *Spn* infection is critical for synergistic mortality. Thus, *Spn* infection 3 days after SARS-CoV-2 increased mortality, whereas *Spn* infection 3 days before SARS-CoV-2 did not. Furthermore, superinfection with a PLY-deficient *Spn* strain further increased mortality and pathology and promoted proinflammatory cytokine expression and the accumulation of neutrophils and macrophages in the lung. These results indicate that *Spn* superinfection aggravates SARS-CoV-2-induced lung disease, whereas PLY attenuates disease severity.

## 2. Materials and Methods

### 2.1. Mice

Male and female adult C57BL/6 mice (originally obtained from The Jackson Laboratory, Bar Harbor, ME, and bred in approved facilities at the University of Texas Health Science Center at San Antonio) over 5 months of age (mo) were used for these studies. Age- and sex-matched controls were used for all experiments.

### 2.2. Bacterial and Viral Strains

SARS-CoV-2 MA10 was propagated in VeroE6-TMPRSS2 cells. Briefly, VeroE6-TMPRSS2 cells were plated in a T175 flask containing DMEM supplemented with 10% FBS and allowed to reach 90% confluence. Cells were inoculated with SARS-CoV-2 MA10 at a multiplicity of infection (MOI) of 0.001 or 0.01 and monitored for cytopathic effect. At 48–72 h post-infection, the supernatant was collected, centrifuged for 10 min at 1000 *g*, aliquoted, and stored at −80 °C. Virus concentration (PFU/mL) was determined by plaque assay. The TIGR4 (serotype 4 clinical isolate) strain of *S. pneumoniae* and a pneumolysin-deficient strain of *Spn* (*Spn^Δply^*) [27] were kindly provided by Dr. Carlos Orihuela (University of Alabama at Birmingham, Birmingham, AL). Bacterial concentration (CFU/mL) was determined by serial dilution on blood agar plates.

### 2.3. Viral and Bacterial Infections in Mice

For SARS-CoV-2 infections, mice were anesthetized with 90 mg/kg ketamine and 10 mg/kg xylazine via intraperitoneal (i.p.) injection. Mice were infected intranasally (i.n.) with 5 × 10^5^ PFU of MA10 in 50 µL, unless otherwise indicated. For *Spn* infection, mice were anesthetized with ketamine/xylazine and infected i.n. with the indicated dose of wild-type (WT) *Spn* or a pneumolysin-deficient strain (*Spn^Δply^*) in 25 µL. Control mice were anesthetized and injected i.n. with PBS. Body weight was monitored daily after infection. Death was not used as an experimental endpoint. Mice were considered moribund and were euthanized with an isoflurane overdose if they lost more than 20% of body weight and were counted as dead for the survival experiments. Control mice with either SARS-CoV-2 or *Spn* mono-infection were anesthetized and mock-infected at the time of sequential infection.

### 2.4. Bacterial and Viral Loads

To quantify bacterial load, the inferior lobe of the infected lung was homogenized in 1 mL of PBS, serially diluted, and plated on blood agar. Bacterial colonies were counted after 24 h at 37 °C. To quantify viral titer, the middle lung lobe was homogenized in 1 mL of lysis buffer, and total RNA was extracted using the Quick-RNA miniprep kit (Zymo Research, Irvine, CA, USA). cDNA synthesis and real-time PCR were performed as previously described using Power SYBR Green master mix (Applied Biosystems, Carlsbad, CA, USA). A standard curve was generated from a diluted plasmid containing the SARS-CoV-2 RBD and used to convert Ct values to viral RNA load, as previously described [33]. To assess active SARS-CoV-2 replication, real-time PCR for subgenomic N RNA was performed, as described [34].

### 2.5. Histopathology

Mice were euthanized, and the lungs and hearts were removed as a single block and fixed in 10% neutral buffered formalin (Thermo Fisher Scientific, Waltham, MA, USA) for 24 h. Tissues were processed, embedded in paraffin, and sectioned at 5 µm for hematoxylin and eosin (H&E, Vector Laboratories, Newark, CA, USA) staining and pathology scoring. Brightfield images were acquired on a Keyence BZ-X800 microscope (Keyence Corporation, Itasca, IL, USA) at 10× magnification.

### 2.6. Gross Pathology and Histological Scoring

Gross lung lesions were scored at necropsy on the following scale: 0 = no noticeable discoloration or lesions; 1 = lesions or discoloration affecting <25% of the lung area; 2 = 25–50% discoloration; 3 = 50–75% discoloration; 4 = >75% discoloration.

Formalin-fixed, paraffin-embedded lung tissues were sectioned at 5 µm and stained with H&E for pathology scoring, as described [35]. Lesions were scored in the following categories: bronchiolitis, thrombosis, fibrin, necrosis, interstitial pneumonia, edema, and hemorrhage, on a scale of 0–4; 0 = no lesion, 1 = minimum, 2 = mild, 3 = moderate, and 4 = severe. The sum of all lesion category scores for each mouse was used for data plotting and analysis. Scores from 3 slides per animal in each group were averaged, and statistical significance was assessed using Welch’s *t*-test or the nonparametric Mann–Whitney test.

### 2.7. Immunofluorescence

Mice were euthanized, and lungs were fixed in 4% paraformaldehyde for 24 h, incubated in 30% sucrose for 24 h, and embedded in Tissue-Tek Optimal Cutting Temperature (OCT, Sakura Finetek USA, Torrance, CA, USA) compound. Sections were cut at 10 µm on a Cryostar NX0 cryostat (Thermo Fisher Scientific, Waltham, MA, USA), adhered to Superfrost Plus slides, blocked for 1 h at room temperature (RT) with 1% BSA, 10% goat serum, and 0.3% Triton X-100 (MilliporeSigma, St. Louis, MO, USA) in PBS, incubated with primary antibodies for 24 h at 4 °C, washed in PBS, and incubated with labeled secondary antibodies for 1.5 h at RT. Antibodies were purchased from BioLegend (San Diego, CA, USA). Slides were washed in PBS and stained with DAPI (Thermo Fisher Scientific, Waltham, MA, USA) for 10 min at RT. After a final wash, slides were mounted with ProLong Gold Antifade (Thermo Fisher Scientific, Waltham, MA, USA) and imaged on a Keyence BZ-X800 microscope. Fluorescent images were analyzed in QuPath v0.7.0 [36] for automated cell detection and quantification. Region-of-interest images were imported into QuPath, and nuclear counterstaining (DAPI) was used to identify individual cell nuclei with QuPath’s built-in cell detection algorithm and to delineate cell boundaries using the default cell expansion setting. Detection parameters were held constant across all samples. Mean cytoplasmic fluorescence intensity was used to distinguish positive and negative populations with a fixed intensity threshold. For each experimental group, three to four mice were analyzed, with 3–5 fields of view imaged and quantified per mouse.

### 2.8. Real-Time RT-PCR Analysis

RNA was isolated from lungs of infected mice using the Quick-RNA miniprep kit (Zymo Research, Irvine, CA, USA). cDNA synthesis and real-time PCR were performed as described previously using Power SYBR Green master mix (Applied Biosystems, Carlsbad, CA, USA). Relative mRNA expression was determined using the 2^−ΔΔCt^ method and normalized to HPRT. Primers are listed in Appendix A, Table A1.

### 2.9. Statistical Analysis

All statistics were calculated using GraphPad Prism (version 11). Statistical significance was assessed using two-way ANOVA, the Mann–Whitney test, Welch’s *t*-test or unpaired *t*-test, as appropriate. Survival was assessed using the log-rank (Mantel–Cox) test. Not significant, *p* > 0.05 (ns); *p* < 0.05 (*); *p* < 0.01 (**); *p* < 0.001 (***); *p* < 0.0001 (****). Data are presented as means ± SEM unless otherwise specified.

## 3. Results

### 3.1. S. pneumoniae Superinfection Increases SARS-CoV-2-Induced Mortality

To investigate a potential synergy between SARS-CoV-2 and *Spn* infections, we first established a non-lethal *Spn* infection model. We infected C57BL/6 (WT) mice with 5 × 10^3^, 5 × 10^4^, or 5 × 10^5^ CFU of the TIGR4 strain of *Spn* via the intranasal route (i.n.) (Figure 1A). None of the doses resulted in mortality, with only limited body weight loss (Figure 1B). While the lower doses of *Spn* resulted in minimal lung inflammation (Figure 1C) and mild pathology scores (Figure 1D), the highest dose of *Spn* caused significant lung discoloration, indicating moderate-to-severe lung damage (Figure 1C,D). These results confirm that *Spn* mono-infection within the dose range we tested promotes lung pathology without causing significant body weight loss or mortality in mice. Therefore, for subsequent experiments, we chose 3 × 10^5^ CFU of *Spn*, which alone causes significant but not severe lung damage.

To test whether prior *Spn* infection increases susceptibility to subsequent SARS-CoV-2 infection, we infected mice i.n. with 3 × 10^5^ CFU of *Spn*, followed 3 days later by a non-lethal (5 × 10^5^ PFU) infection with the mouse-adapted strain of SARS-CoV-2 (MA10) (Figure 1E). While MA10 infection alone caused up to 20% body weight loss by day 4 post-infection (dpi), prior *Spn* infection did not exacerbate weight loss after MA10 infection (Figure 1F). No mortality was observed in any experimental group. These results suggest that prior *Spn* infection does not predispose mice to more severe COVID-19 disease.

To test whether follow-up *Spn* superinfection can promote SARS-CoV-2-induced pathology, we infected WT mice with 5 × 10^5^ PFU of MA10 and, 3 days later, with 3 × 10^5^ CFU of *Spn* (Figure 1G). Although *Spn* superinfection did not significantly affect SARS-CoV-2-induced body weight loss, we observed significantly increased mortality in the combined MA10 + *Spn*-infected group, with a more pronounced effect in male mice (Figure 1H,I). These data indicate that *Spn* superinfection during the peak of SARS-CoV-2 infection increases mortality.

### 3.2. Spn Superinfection Increases SARS-CoV-2 Replication, Lung Pathology, and Proinflammatory Immune Responses

To further characterize how *Spn* infection exacerbates lung pathology in SARS-CoV-2-infected mice, we infected WT mice with 5 × 10^5^ PFU of MA10, followed by 3 × 10^5^ PFU of *Spn* at 3 dpi, and analyzed lung pathology at 6 dpi (Figure 2A). We used female mice in these experiments to limit mortality after combined infection and to collect lung tissue for analysis at later time points. Superinfected mice exhibited more severe lung discoloration and higher gross pathology scores (Figure 2B,C), along with increased histopathology scores (Figure 2D,E). Immunofluorescence analysis revealed a higher number of neutrophils (Ly6G^+^) in MA10 + *Spn*-infected mice compared with MA10-only-infected mice (Figure 2F,G). In contrast, macrophage (CD68^+^) and T cell (CD4^+^) numbers did not differ significantly between groups (Figure 2F,G). Strikingly, superinfected mice had significantly higher lung SARS-CoV-2 titers, measured by spike RNA levels, as well as higher SARS-CoV-2 genomic and subgenomic N RNA levels, indicating increased viral replication (Figure 2H).

Analysis of cytokine expression at 6 dpi revealed increased IFN-β levels and a trend toward higher expression of the proinflammatory cytokines IFN-γ, IL-6, and IL-17A, as well as the IL-10 family cytokines IL-10 and IL-22 in the lungs of MA10 + *Spn*-infected mice compared with those infected with MA10 only (Figure 2I). To test whether an increased number of neutrophils is linked to elevated expression of neutrophil-recruiting chemokines, we measured expression of CXCL1, CXCL2, and CXCL12 in the lungs. We detected increased expression of CXCL2 and a trend toward increased CXCL1 expression in coinfected lungs (Figure 2J), which corresponded to an increased number of neutrophils (Figure 2F). Thus, these data suggest that subsequent *Spn* infection increases SARS-CoV-2 replication and lung immunopathology, associated with increased neutrophil recruitment and elevated expression of proinflammatory cytokines and neutrophil-recruiting chemokines.

### 3.3. Pneumolysin Reduces Mortality in SARS-CoV-2 + Spn Superinfection

Next, we sought to identify factors that drive pathogenic immune responses during SARS-CoV-2 + *Spn* superinfection. Pneumolysin (PLY), a pore-forming toxin produced by *Spn*, is a critical *Spn* virulence factor [21,23]. It can create transmembrane pores in host cells, damaging the plasma membrane and disrupting cellular homeostasis [23,31]. Although PLY primarily facilitates *Spn* colonization and survival, it also strongly triggers and modulates innate and adaptive immune responses against bacterial infection [24,28,37].

To evaluate the role of PLY in increased mortality following SARS-CoV-2 + *Spn* superinfection, we infected WT mice with MA10 and, at 3 dpi, infected them with either wild-type *Spn* or a PLY-deficient strain of *Spn* (*Spn^Δply^*) (Figure 3A). Previous studies showed that WT mice infected with *Spn^Δply^* alone had limited colonization and no weight loss or mortality [27,38]. Surprisingly, mice infected with *Spn^Δply^* 3 days after MA10 infection exhibited greater body weight loss than those infected with the wild-type strain of *Spn* and had 100% mortality by 8 dpi (Figure 3B,C). At 8 dpi, lungs of mice infected with *Spn^Δply^* showed severe pathology, with edematous, darkly discolored areas and the highest gross pathology scores (Figure 3D,E). Thus, these results suggest that *Spn* PLY restricts superinfection-driven lung disease.

### 3.4. Pneumolysin Limits Proinflammatory Immune Activation During SARS-CoV-2 + Spn Superinfection Without Affecting Viral or Bacterial Replication

To determine whether increased lung pathology and mortality in *Spn^Δply^* superinfected mice were due to increased viral or bacterial growth or impaired pathogen clearance, we infected WT mice on day 3 after SARS-CoV-2 infection with *Spn* or *Spn^Δply^* and analyzed lungs at 6 dpi (Figure 4A). We found no difference in SARS-CoV-2 viral RNA levels or bacterial growth in the lungs (Figure 4B), suggesting that increased mortality in *Spn^Δply^* superinfected mice is not due to increased pathogen load. Interestingly, we observed a trend toward elevated expression of IFN-γ, IL-10, IL-22, TNF, IL-1β, IL-6, CXCL1, and CXCL2 in a subset of *Spn^Δply^* superinfected mice (Figure 4C), although this difference was not statistically significant because of high variability in the *Spn^Δply^*-infected group. Histological analysis of lungs revealed increased pathology, associated with edema, inflammation, and diffuse alveolar damage (Figure 4D,E). Immunofluorescence analysis revealed an increased number of CD68^+^ macrophages and Ly6G^+^ neutrophils but not CD4^+^ T cells in MA10 + *Spn^Δply^*-infected mice compared to MA10 + *Spn*-infected mice (Figure 4F,G). These results indicate that PLY limits lung disease in SARS-CoV-2 + *Spn* superinfection potentially by attenuating macrophage- and neutrophil-associated proinflammatory immune responses.

## 4. Discussion

In this study, we demonstrate that *Spn* superinfection exacerbates SARS-CoV-2-induced lung disease in WT mice. Increased susceptibility after *Spn* superinfection is associated with elevated viral replication and lung pathology, greater neutrophil accumulation, and increased expression of IFN-β and the neutrophil-recruiting chemokine CXCL2. *Spn* PLY attenuates disease severity by potentially inhibiting macrophage- and neutrophil-mediated lung immunopathology. These results highlight the critical role of PLY in mitigating lung immunopathology caused by SARS-CoV-2 and *Spn* superinfection.

Mouse models of SARS-CoV-2 and *Spn* co-infection are essential for understanding synergistic disease mechanisms. WT mice do not develop disease after infection with human SARS-CoV-2 isolates due to incompatibility between the SARS-CoV-2 Spike protein and mouse ACE2 [39,40]. Therefore, we used a mouse-adapted MA10 strain, which can induce lung pathology in WT mice [32]. Our results suggest that *Spn* promoted lung pathology and mortality when administered after MA10 infection.

Two previous reports used transgenic mice expressing human ACE2 under the K18 promoter (K18-hACE2-Tg) to study SARS-CoV-2 and *Spn* co-infection [18,19]. Although both studies showed that *Spn* co-infection can increase mortality in SARS-CoV-2-infected K18-hACE2-Tg mice, the impact of *Spn* administration on mortality and lung pathology varied between studies, depending on the dose, *Spn* strain, and timing of administration. Thus, Barman et al. [18] showed that *Spn* (A66.1 serotype 3, 10^3^ CFU) administered 2 days before or on day 6 after SARS-CoV-2 infection (WA1/2020, 10^3^ PFU) increased lung pathology and mouse mortality. In contrast, Smith et al. [19] showed that increased mortality occurred only when *Spn* (D39 serotype 2, 10^3^ CFU) was administered on day 5 or 7, but not on day 3 after low-dose SARS-CoV-2 (WA1/2020, 250 PFU) infection, and that bacterial co-infection did not enhance lung pathology. It is important to note that K18-hACE2-Tg mice are extremely sensitive to SARS-CoV-2 infection due to ectopic expression of hACE2 in the central nervous system neurons and other organs besides the lung [41,42,43,44,45]. The use of a mouse-adapted MA10 strain in WT mice allowed us to utilize higher doses of the virus and study lung pathology, avoiding limitations of K18-hACE2-Tg mice [46]. Our results suggest that *Spn* superinfection on day 3 after a high-dose SARS-CoV-2 infection (5 × 10^5^ PFU) results in higher mortality and lung pathology. In contrast, *Spn* administration 3 days before SARS-CoV-2 infection did not increase mortality, suggesting that early antibacterial immune responses to *Spn* could contribute to protection against subsequent SARS-CoV-2 infection, which future studies can further investigate. Furthermore, *Spn* superinfection resulted in higher mortality in males compared to females, consistent with increased sensitivity of male mice to SARS-CoV-2 infection [42,46].

The mechanisms that contribute to disease severity in combined bacterial and viral co-infection with SARS-CoV-2 are complex and vary by pathogen. Other studies in animal models have demonstrated synergistic lethality between SARS-CoV-2 and *Staphylococcus aureus* [47], *Klebsiella pneumoniae* [35] and influenza virus [8,9,48,49]. Increased proinflammatory signaling, leading to a cytokine storm after combined infection, may contribute to synergistic lethality [9,18]. Additionally, increased recruitment of neutrophils and inflammatory monocytes, along with loss of alveolar macrophages, is associated with increased lung pathology in SARS-CoV-2 co-infection [35]. A recent study showed that ACE2-expressing epithelial cells, club cells, and macrophages distinctively contribute to proinflammatory cytokine and chemokine production, neutrophil recruitment, and lung pathology in mice [33]. Consistent with these studies, we detected increased mRNA levels of IFN-β and the neutrophil-recruiting chemokine CXCL2 in the lung after MA10 + *Spn* superinfection, which were associated with increased neutrophil numbers in the lung. Other proinflammatory cytokines and chemokines, such as IL-6, TNF, IL-1β, IFN-γ, and CXCL1, showed a trend toward elevated expression, but differences were not statistically significant because of high variability and a limited sample size. Further analysis of protein expression and kinetics is needed to characterize proinflammatory responses in MA10 + *Spn* superinfection. It also remains to be determined whether selectively targeting these components could ameliorate lung pathology and mortality after SARS-CoV-2 + *Spn* superinfection.

PLY is a key pneumococcal virulence factor that plays a dual role during *Spn* infection by activating both proinflammatory signaling and anti-inflammatory mechanisms [20,21,22,23]. PLY is a pore-forming toxin that disrupts the host cell barrier to facilitate bacterial colonization [20,21,22]. Our results with a PLY-deficient *Spn* strain indicate that PLY contributes to protection against SARS-CoV-2 + *Spn* superinfection by reducing lung pathology and mortality. Interestingly, PLY deficiency did not affect viral or bacterial titers, indicating that increased lung pathology after *Spn* superinfection is not due to viral-mediated cytotoxicity, but rather due to dysregulated immune responses. During mono-infection, a *Spn^Δply^* mutant strain typically fails to establish infection and is rapidly cleared by the host [27]. However, a preceding infection, such as a high dose of MA10 SARS-CoV-2, may compromise cellular membrane integrity, allowing the *Spn^Δply^* mutant to colonize the host just as effectively as the WT strain. This may explain why we observed no differences in bacterial titers in the lungs of mice infected with WT *Spn* or *Spn^Δply^* after SARS-CoV-2 infection. Furthermore, the numbers of Ly6G^+^ neutrophils and CD68^+^ macrophages were increased in the lungs of SARS-CoV-2 + *Spn^Δply^* co-infected mice, compared to the WT *Spn* co-infected mice, indicating anti-inflammatory effects of PLY. Interestingly, a previous study showed that administration of recombinant PLY or isogenic PLY complementation promoted CXCL2 and IL-1β expression and neutrophil recruitment to the upper respiratory tract in neonatal mice [27], indicating that pro- and anti-inflammatory effects of PLY can be distinct in neonatal and adult mice.

Beyond barrier disruption, PLY expression by *Spn* in SARS-CoV-2-infected lungs may promote macrophage and neutrophil death, causing severe localized tissue damage. In contrast, the *Spn^Δply^* mutant may allow immune cell survival, provoking a sustained cytokine and chemokine response that ultimately drives higher host mortality than PLY-expressing *Spn* during superinfection. Our findings suggest that increased lethality in SARS-CoV-2 + *Spn^Δply^* superinfection results from host immunopathology rather than from off-target effects of the *Spn^Δply^* mutant. This conclusion is further supported by previous mono-infection and complementation studies, which thoroughly characterized the *Spn^Δply^* mutant used in this study and ruled out off-target genetic artifacts [27].

Consistent with our results, a previous study showed that PLY inhibits alveolar macrophage apoptosis and reduces the influx of CD11b^+^Ly6C^hi^ inflammatory monocytes in the lungs of mice coinfected with influenza and *Spn* [38]. Additionally, PLY can inhibit proinflammatory cytokine production by human monocyte-derived macrophages in vitro and in vivo in WT mice after *Spn* infection [37]. The anti-inflammatory effects of PLY are mediated by binding to MRC-1 (CD206) on macrophages and dendritic cells, supporting anti-inflammatory M2 macrophage polarization and function [28,37,50]. MRC-1, a C-type lectin receptor, also acts as a carbohydrate-binding pattern recognition receptor that can interact with the heavily glycosylated surface spike glycoprotein of SARS-CoV-2 [51]. Alveolar M2 macrophages highly express CD206, and a deficit in tissue-repairing M2 responses correlates with worsened lung pathology in COVID-19 patients [52,53,54,55]. In contrast, an influx of neutrophils and pro-inflammatory, monocyte-derived macrophages, along with enhanced M1 macrophage responses, supports unmitigated local alveolitis and systemic hyperinflammation [54,56,57,58,59]. Therefore, we speculate that PLY mitigates lung pathology in SARS-CoV-2 + *Spn* co-infection by attenuating macrophage/neutrophil-mediated immunopathology. The mechanistic link between PLY and macrophage and neutrophil function in mitigating immunopathology will require further analysis of macrophage subsets and their functional states, as well as measurement of neutrophil extracellular trap formation. In addition to alveolar macrophages, interstitial macrophages, including nerve- and airway-associated interstitial macrophages, participate in the regulation of lung pathology during SARS-CoV-2 infection [60,61]. Whether PLY can interact with these macrophages and affect their responses during SARS-CoV-2 + *Spn* superinfection remains to be determined.

*Spn* infection impairs host immunity to SARS-CoV-2, including mucosal IgA and T cell responses [62]. Although PLY can affect CD4 T cell migration [63], its role in regulating adaptive immune responses during SARS-CoV-2 + *Spn* superinfection remains unclear.

## 5. Conclusions

In conclusion, using a SARS-CoV-2 and *Spn* co-infection model in WT mice, we demonstrated that sequential *Spn* infection exacerbates SARS-CoV-2-induced mortality. Our results show that *Spn* infection increases SARS-CoV-2 replication, lung pathology, and neutrophil accumulation. *Spn* PLY plays an important role in mitigating superinfection-induced lung pathology without affecting viral or bacterial titers. Thus, infection with *Spn^Δply^* exacerbates SARS-CoV-2-induced lung immunopathology, increases proinflammatory cytokine expression, and increases macrophage and neutrophil accumulation in the lung. This work expands our understanding of how *Spn* co-infection contributes to SARS-CoV-2-induced lung disease and highlights PLY as a potential target to mitigate lung immunopathology.

## Figures and Tables

**Figure 1:**
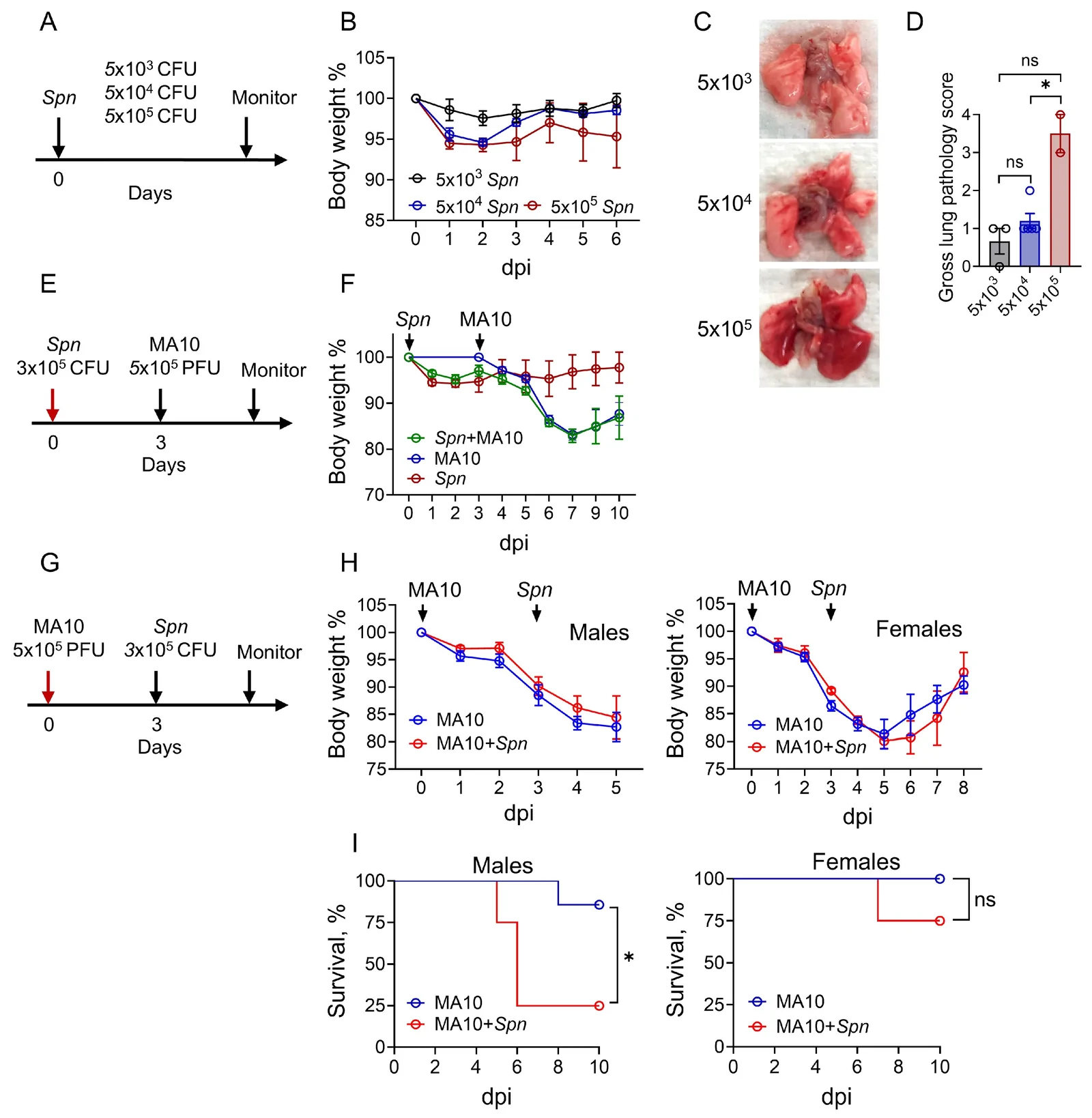
*S. pneumoniae* superinfection exacerbates SARS-CoV-2-induced disease. (**A**–**D**). *Spn* infection induces lung pathology without mortality. Male (6–7 mo) and female (5–6 mo) C57BL/6 (WT) mice were intranasally (i.n.) infected with the indicated doses of *Spn*. (**A**) Experimental scheme. (**B**) Body weight loss (*n* = 4–6). (**C**) Representative lung images at 6 dpi. (**D**) Gross lung pathology scores at 6 dpi (*n* = 2–5). (**E**,**F**). SARS-CoV-2 administered 3 days after *Spn* infection does not increase mortality. WT female mice (5 mo) were i.n. infected with 3 × 10^5^ CFU of *Spn* or mock-infected. On 3 dpi, they were i.n. infected with 5 × 10^5^ PFU of mouse-adapted SARS-CoV-2 (MA10). (**E**) Experimental scheme. (**F**) Body weight loss (*n* = 4–7). (**G**–**I**). *Spn* infection administered 3 days after SARS-CoV-2 infection promotes mortality. WT mice were i.n. infected with 5 × 10^5^ PFU of MA10. On 3 dpi, they were i.n. infected with 3 × 10^5^ CFU of *Spn* or mock-infected. (**G**) Experimental scheme. Body weight loss and survival of male (6 mo) (**H**) or female (5 mo) (**I**) mice (*n* = 4–5). Data are presented as mean ± SEM. Survival data were analyzed using the log-rank (Mantel–Cox) test. All other data were analyzed by the nonparametric Mann–Whitney test. ns—not significant, * *p* < 0.05.

**Figure 2:**
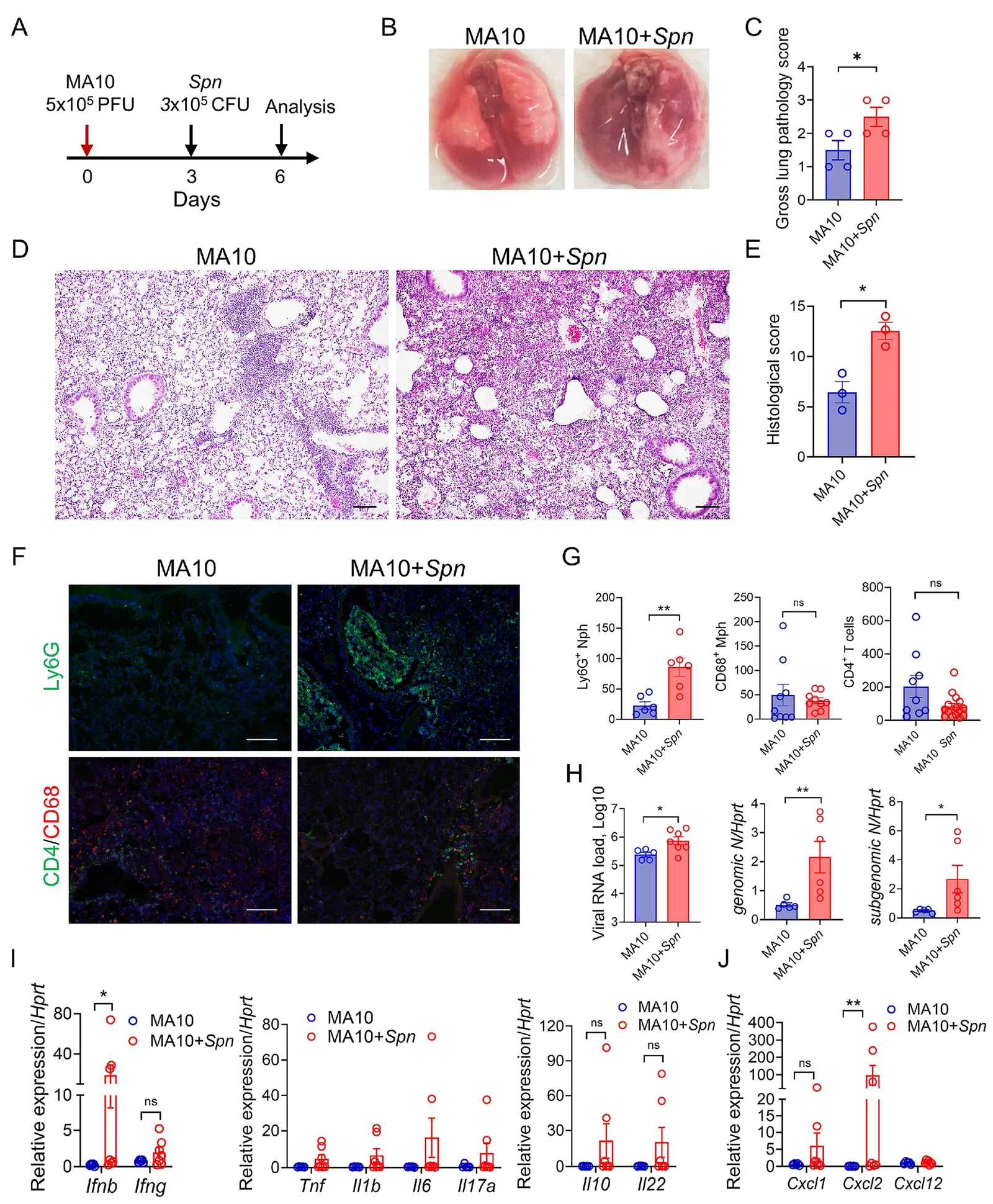
*S. pneumoniae* superinfection increases viral replication, lung pathology, and neutrophil recruitment. WT female mice (6 mo) were infected with SARS-CoV-2 MA10, followed by *Spn* at 3 dpi, and euthanized at 6 dpi. (**A**) Experimental scheme. (**B**) Representative lung images at 6 dpi. (**C**) Gross pathology scores (*n* = 4). (**D**) Representative H&E images (10×). (**E**) Histological scores (*n* = 3). (**F**) Representative immunofluorescence of neutrophils (Ly6G^+^), macrophages (CD68^+^), and T cells (CD4^+^). (**G**) Quantification of Ly6G^+^ neutrophils (Nph), CD68^+^ macrophages (Mph), and T cells (CD4^+^) in the lung, per field of view (*n* = 4). (**H**) SARS-CoV-2 lung viral RNA load (measured by spike RNA RT-PCR) and SARS-CoV-2 replication (measured by RT-PCR for genomic/subgenomic N RNA) (*n* = 5–7). (**I**) Expression of interferons, proinflammatory cytokines, and IL-10 family cytokines was measured by RT-PCR (*n* = 5–7). (**J**) Chemokine expression was measured by RT-PCR (*n* = 5–7). Data are presented as mean ± SEM. Data were analyzed by Welch’s *t*-test (**C**,**E**,**G**) or by the nonparametric Mann–Whitney test (**H**–**J**). ns—not significant, * *p* < 0.05, ** *p* < 0.01. Scale bars are 100 µm.

**Figure 3:**
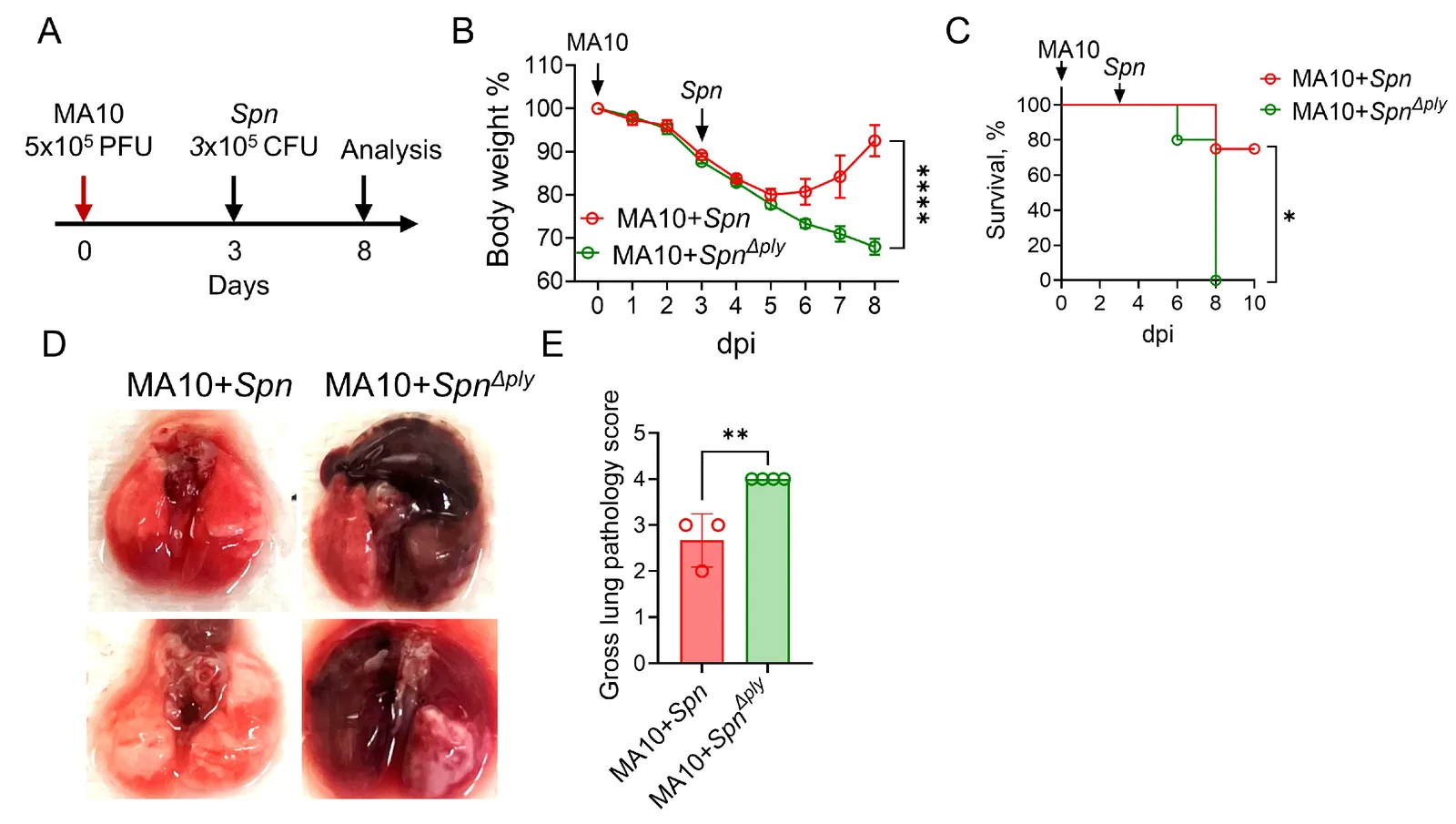
Superinfection with PLY-deficient *S. pneumoniae* increases mortality after SARS-CoV-2 infection. WT female mice (5 mo) were infected with SARS-CoV-2 MA10, followed by infection with *Spn* or *Spn^Δply^*. (**A**) Experimental scheme. (**B**) Body weight (*n* = 4–5). (**C**) Survival (*n* = 4–5). (**D**) Representative lung images at 8 dpi. (**E**) Gross pathology scores at 8 dpi (*n* = 3–4). Data are presented as mean ± SEM. Body weight data were analyzed by two-way ANOVA; survival data were analyzed by the log-rank (Mantel–Cox) test; and all other data were analyzed by unpaired *t*-test. * *p* < 0.05; ** *p* < 0.01; **** *p* < 0.0001.

**Figure 4:**
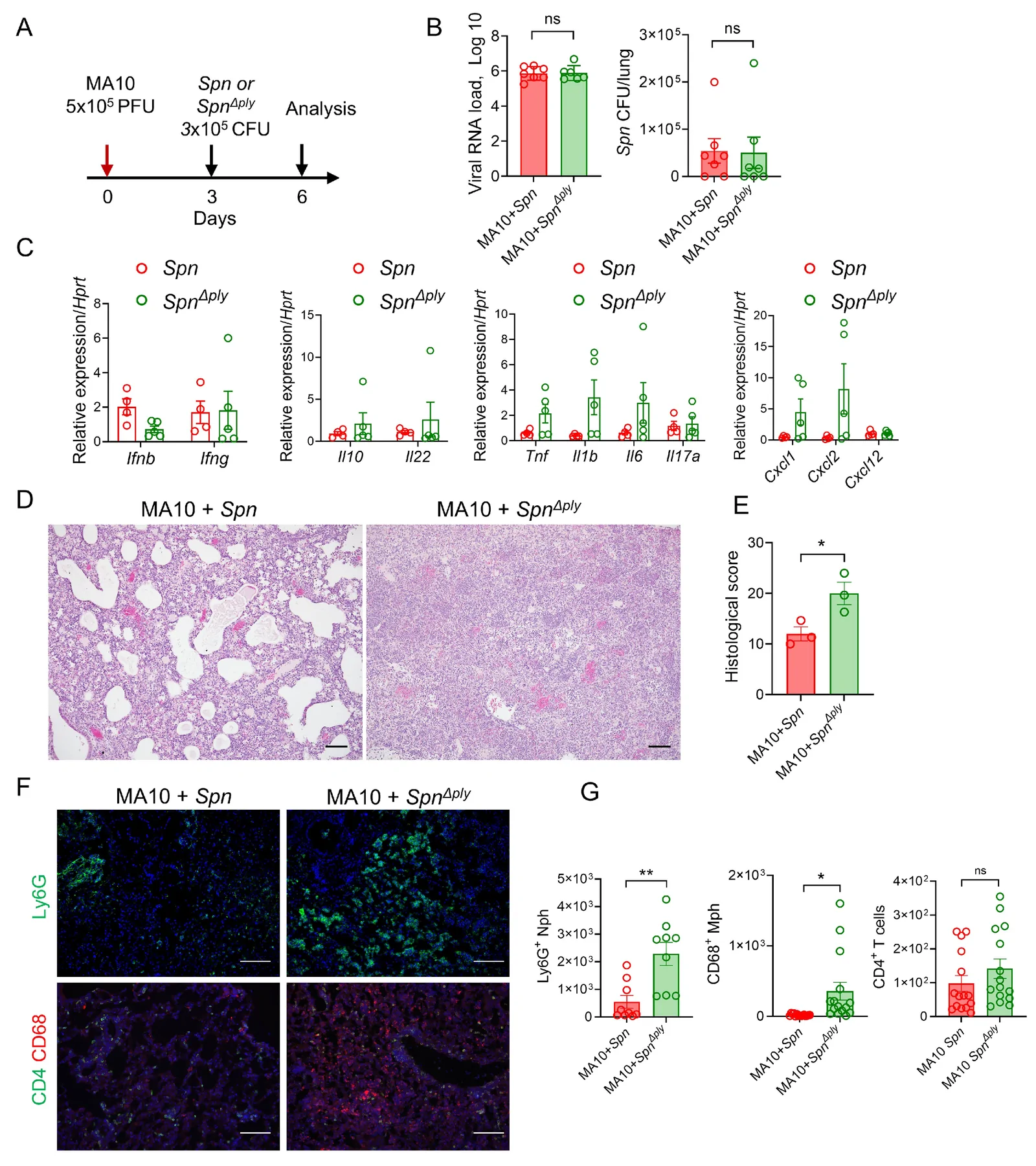
Pneumolysin restricts proinflammatory immune activation and immune-mediated pathology during SARS-CoV-2 + *Spn* superinfection. WT female mice (6 mo) were infected with SARS-CoV-2 MA10, followed by *Spn* or *Spn^Δply^* at 3 dpi, and euthanized at 6 dpi. (**A**) Experimental scheme. (**B**) SARS-CoV-2 viral RNA load and *Spn* CFU in the lungs (*n* = 7). (**C**) Cytokine and chemokine expression in the lungs measured by RT-PCR (*n* = 4–5). (**D**) Representative H&E images (10×). (**E**) Histological scores (*n* = 3). (**F**) Immunofluorescent staining for Ly6G^+^ neutrophils (Nph), CD68^+^ macrophages (Mph), and CD4^+^ T cells. (**G**) Quantification of Ly6G^+^, CD68^+^, and CD4^+^ cells (*n* = 4). Data are presented as mean ± SEM. Data were analyzed using the nonparametric Mann–Whitney test (**B**,**C**) or by Welch’s *t*-test (**E**,**G**). ns—not significant; * *p* < 0.05; ** *p* < 0.01. Scale bars are 100 µm.

## Data Availability

The authors confirm that the data supporting the findings of this study are available within the article. Any additional information required to reanalyze the data reported in this paper is available from the lead contact upon request.

## Abbreviations

The following abbreviations are used in this manuscript:

*Spn*—*Streptococcus pneumoniae*; PLY—pneumolysin; nph—neutrophil; mph—macrophage; dpi—day post infection; mo—month of age; i.n.—intranasal; i.p.—intraperitoneal; CFU—colony-forming units; PFU—plaque-forming units, MOI—multiplicity of infection; PBS—phosphate-buffered saline; hACE2—human angiotensin-converting enzyme 2; MRC-1—macrophage mannose receptor 1; RBD—receptor binding domain; OCT—Optimal Cutting Temperature Compound; DAPI—4′,6-diamidino-2-phenylindole; IACUC—Institutional Animal Care and use Committee.

## Appendix A

**Table A1:** Primers for quantitative PCR.

Primer	Sequence
Ifng FW	TCAAGTGGCATAGATGTGGAAGAA
Ifng RV	TGGCTCTGCAGGATTTTCATG
Tnf FW	ACGGCATGGATCTCAAAGAC
Tnf RV	AGATAGCAAATCGGCTGACG
Ifnb FW	ATGAGTGGTGGTTGCAGGC
Ifnb RV	TGACCTTTCAAATGCAGTAGATTCA
Il6 FW	ACAAGTCGGAGGCTTAATTACACAT
Il6 RV	AATCAGAATTGCCATTGCACAA
Il1B FW	TGGACCTTCCAGGATGAGGACA
Il1B RV	GTTCATCTCGGAGCCTGTAGTG
Il10 FW	CGGGAAGACAATAACTGCACCC
Il10 RV	CGGTTAGCAGTATGTTGTCCAGC
Il17a FW	CAGACTACCTCAACCGTTCCAC
Il17a RV	TCCAGCTTTCCCTCCGCATTGA
Il22 FW	TCCGAGGAGTCAGTGCTAAA
Il22 RV	AGAACGTCTTCCAGGGTGAA
mHprt FW	CTGGTGAAAAGGACCTCTCGAAG
mHprt RV	CCAGTTTCACTAATGACACAAACG
RBD (Spike protein) FW	CAATGGTTTAACAGGCACAGG
RBD (Spike protein) RV	CTCAAGTGTCTGTGGATCACG
Genomic N protein FW	AAATTTTGGGGACCAGGAAC
Genomic N protein RV	TGGCAGCTGTGTAGGTCAAC
Subgenomic N protein FW	CCAGGTAACAAACCAACCAACTTTCG
Subgenomic N protein RV	GGTTACTGCCAGTTGAATCTGAGG
Cxcl1 FW	GCTGGGATTCACCTCAAGAA
Cxcl1 RV	TGGGGACACCTTTTAGCATC
Cxcl2 FW	CCTGGTTCAGAAAATCATCCA
Cxcl2 RV	CTTCCGTTGAGGGACAGC
Cxcl12 FW	CTGCATCAGTGACGGTAAACC
Cxcl12 RV	CAGCCGTGCAACAATCTGAA

## References

[1] Langford B.J., So M., Raybardhan S., Leung V., Westwood D., MacFadden D.R., Soucy J.R., Daneman N. (2020). Bacterial co-infection and secondary infection in patients with COVID-19: A living rapid review and meta-analysis. Clin. Microbiol. Infect..

[2] Suleiman A.S., Islam M.A., Akter M.S., Amin M.R., Werkneh A.A., Bhattacharya P. (2023). A meta-meta-analysis of co-infection, secondary infections, and antimicrobial resistance in COVID-19 patients. J. Infect. Public Health.

[3] Musuuza J.S., Watson L., Parmasad V., Putman-Buehler N., Christensen L., Safdar N. (2021). Prevalence and outcomes of co-infection and superinfection with SARS-CoV-2 and other pathogens: A systematic review and meta-analysis. PLoS ONE.

[4] Alhumaid S., Al Mutair A., Al Alawi Z., Alshawi A.M., Alomran S.A., Almuhanna M.S., Almuslim A.A., Bu Shafia A.H., Alotaibi A.M., Ahmed G.Y. (2021). Coinfections with Bacteria, Fungi, and Respiratory Viruses in Patients with SARS-CoV-2: A Systematic Review and Meta-Analysis. Pathogens.

[5] Wu H.Y., Chang P.H., Chen K.Y., Lin I.F., Hsih W.H., Tsai W.L., Chen J.A., Lee S.S. (2022). Coronavirus disease 2019 (COVID-19) associated bacterial coinfection: Incidence, diagnosis and treatment. J. Microbiol. Immunol. Infect..

[6] Calvo-Silveria S., Fernandez-Delgado L., Gonzalez-Diaz A., Espana-Bonilla R., Calatayud L., Niubo J., Marti S., Dominguez M.A., Camara J., Ardanuy C. (2025). SARS-CoV-2 and *Streptococcus pneumoniae* colonization and disease: An observational study in adults. Front. Cell. Infect. Microbiol..

[7] Langford B.J., So M., Simeonova M., Leung V., Lo J., Kan T., Raybardhan S., Sapin M.E., Mponponsuo K., Farrell A. (2023). Antimicrobial resistance in patients with COVID-19: A systematic review and meta-analysis. Lancet Microbe.

[8] Yan X., Li K., Lei Z., Luo J., Wang Q., Wei S. (2023). Prevalence and associated outcomes of coinfection between SARS-CoV-2 and influenza: A systematic review and meta-analysis. Int. J. Infect. Dis..

[9] Achdout H., Vitner E.B., Politi B., Melamed S., Yahalom-Ronen Y., Tamir H., Erez N., Avraham R., Weiss S., Cherry L. (2021). Increased lethality in influenza and SARS-CoV-2 coinfection is prevented by influenza immunity but not SARS-CoV-2 immunity. Nat. Commun..

[10] Russell C.D., Fairfield C.J., Drake T.M., Turtle L., Seaton R.A., Wootton D.G., Sigfrid L., Harrison E.M., Docherty A.B., de Silva T.I. (2021). Co-infections, secondary infections, and antimicrobial use in patients hospitalised with COVID-19 during the first pandemic wave from the ISARIC WHO CCP-UK study: A multicentre, prospective cohort study. Lancet Microbe.

[11] Westblade L.F., Simon M.S., Satlin M.J. (2021). Bacterial Coinfections in Coronavirus Disease 2019. Trends Microbiol..

[12] Amin-Chowdhury Z., Aiano F., Mensah A., Sheppard C.L., Litt D., Fry N.K., Andrews N., Ramsay M.E., Ladhani S.N. (2021). Impact of the Coronavirus Disease 2019 (COVID-19) Pandemic on Invasive Pneumococcal Disease and Risk of Pneumococcal Coinfection with Severe Acute Respiratory Syndrome Coronavirus 2 (SARS-CoV-2): Prospective National Cohort Study, England. Clin. Infect. Dis..

[13] Sender V., Hentrich K., Henriques-Normark B. (2021). Virus-Induced Changes of the Respiratory Tract Environment Promote Secondary Infections with *Streptococcus pneumoniae*. Front. Cell. Infect. Microbiol..

[14] Teklemariam A.D., Hashem A.M., Saber S.H., Almuhayawi M.S., Haque S., Abujamel T.S., Harakeh S. (2023). Bacterial co-infections and antimicrobial resistance associated with the Coronavirus Disease 2019 infection. Biotechnol. Genet. Eng. Rev..

[15] Buehler P.K., Zinkernagel A.S., Hofmaenner D.A., Wendel Garcia P.D., Acevedo C.T., Gomez-Mejia A., Mairpady Shambat S., Andreoni F., Maibach M.A., Bartussek J. (2021). Bacterial pulmonary superinfections are associated with longer duration of ventilation in critically ill COVID-19 patients. Cell Rep. Med..

[16] Chong W.H., Saha B.K., Ananthakrishnan R., Chopra A. (2021). State-of-the-art review of secondary pulmonary infections in patients with COVID-19 pneumonia. Infection.

[17] Katfy M., Kettani A.E., Katfy K., Diawara I., Nzoyikorera N., Boussetta S., Abdallaoui M.S., Bousfiha A.A. (2025). Coinfection with SARS-CoV-2 among patients with *Streptococcus pneumoniae* in Casablanca. BMC Infect. Dis..

[18] Barman T.K., Singh A.K., Bonin J.L., Nafiz T.N., Salmon S.L., Metzger D.W. (2022). Lethal synergy between SARS-CoV-2 and *Streptococcus pneumoniae* in hACE2 mice and protective efficacy of vaccination. JCI Insight.

[19] Smith A.P., Williams E.P., Plunkett T.R., Selvaraj M., Lane L.C., Zalduondo L., Xue Y., Vogel P., Channappanavar R., Jonsson C.B. (2022). Time-Dependent Increase in Susceptibility and Severity of Secondary Bacterial Infections During SARS-CoV-2. Front. Immunol..

[20] Kadioglu A., Weiser J.N., Paton J.C., Andrew P.W. (2008). The role of *Streptococcus pneumoniae* virulence factors in host respiratory colonization and disease. Nat. Rev. Microbiol..

[21] Rubins J.B., Charboneau D., Paton J.C., Mitchell T.J., Andrew P.W., Janoff E.N. (1995). Dual function of pneumolysin in the early pathogenesis of murine pneumococcal pneumonia. J. Clin. Investig..

[22] Weiser J.N., Ferreira D.M., Paton J.C. (2018). *Streptococcus pneumoniae*: Transmission, colonization and invasion. Nat. Rev. Microbiol..

[23] Pereira J.M., Xu S., Leong J.M., Sousa S. (2022). The Yin and Yang of Pneumolysin During Pneumococcal Infection. Front. Immunol..

[24] McNeela E.A., Burke A., Neill D.R., Baxter C., Fernandes V.E., Ferreira D., Smeaton S., El-Rachkidy R., McLoughlin R.M., Mori A. (2010). Pneumolysin activates the NLRP3 inflammasome and promotes proinflammatory cytokines independently of TLR4. PLoS Pathog..

[25] van Pee K., Mulvihill E., Muller D.J., Yildiz O. (2016). Unraveling the Pore-Forming Steps of Pneumolysin from *Streptococcus pneumoniae*. Nano Lett..

[26] Gonzalez-Juarbe N., Gilley R.P., Hinojosa C.A., Bradley K.M., Kamei A., Gao G., Dube P.H., Bergman M.A., Orihuela C.J. (2015). Pore-Forming Toxins Induce Macrophage Necroptosis during Acute Bacterial Pneumonia. PLoS Pathog..

[27] Zafar M.A., Wang Y., Hamaguchi S., Weiser J.N. (2017). Host-to-Host Transmission of *Streptococcus pneumoniae* Is Driven by Its Inflammatory Toxin, Pneumolysin. Cell Host Microbe.

[28] Subramanian K., Neill D.R., Malak H.A., Spelmink L., Khandaker S., Dalla Libera Marchiori G., Dearing E., Kirby A., Yang M., Achour A. (2019). Pneumolysin binds to the mannose receptor C type 1 (MRC-1) leading to anti-inflammatory responses and enhanced pneumococcal survival. Nat. Microbiol..

[29] Neill D.R., Coward W.R., Gritzfeld J.F., Richards L., Garcia-Garcia F.J., Dotor J., Gordon S.B., Kadioglu A. (2014). Density and duration of pneumococcal carriage is maintained by transforming growth factor beta1 and T regulatory cells. Am. J. Respir. Crit. Care Med..

[30] Ogawa M., Matsuda R., Takada N., Tomokiyo M., Yamamoto S., Shizukuishi S., Yamaji T., Yoshikawa Y., Yoshida M., Tanida I. (2018). Molecular mechanisms of *Streptococcus pneumoniae*-targeted autophagy via pneumolysin, Golgi-resident Rab41, and Nedd4-1-mediated K63-linked ubiquitination. Cell. Microbiol..

[31] Nishimoto A.T., Rosch J.W., Tuomanen E.I. (2020). Pneumolysin: Pathogenesis and Therapeutic Target. Front. Microbiol..

[32] Leist S.R., Dinnon K.H., Schafer A., Tse L.V., Okuda K., Hou Y.J., West A., Edwards C.E., Sanders W., Fritch E.J. (2020). A Mouse-Adapted SARS-CoV-2 Induces Acute Lung Injury and Mortality in Standard Laboratory Mice. Cell.

[33] Todd A.W., Shein S.A., Korchagina A.A., Kudinov V.A., Faz B.N., Barre R.S., Paik R., Collins E.C., Yusoof K.A., Khetarpal I. (2026). Differential effects of SARS-CoV-2-targeted infection of ATII, club cells, and macrophages on lung immunopathology and antiviral responses. Mucosal Immunol..

[34] Mitsui Y., Suzuki T., Kuniyoshi K., Inamo J., Yamaguchi K., Komuro M., Watanabe J., Edamoto M., Li S., Kouno T. (2023). Expression of the readthrough transcript CiDRE in alveolar macrophages boosts SARS-CoV-2 susceptibility and promotes COVID-19 severity. Immunity.

[35] Villalva C., Patil G., Narayanan S.S., Ghimire R., Chanda D., Samarakoon N., Snider T., Ramachandran A., Channappanavar R., More S. (2024). *Klebsiella pneumoniae* co-infection leads to fatal pneumonia in SARS-CoV-2-infected mice. Front. Virol..

[36] Bankhead P., Loughrey M.B., Fernandez J.A., Dombrowski Y., McArt D.G., Dunne P.D., McQuaid S., Gray R.T., Murray L.J., Coleman H.G. (2017). QuPath: Open source software for digital pathology image analysis. Sci. Rep..

[37] Periselneris J., Turner C.T., Ercoli G., Szylar G., Weight C.M., Thurston T., Whelan M., Tomlinson G., Noursadeghi M., Brown J. (2022). Pneumolysin suppresses the initial macrophage pro-inflammatory response to *Streptococcus pneumoniae*. Immunology.

[38] Wolf A.I., Strauman M.C., Mozdzanowska K., Williams K.L., Osborne L.C., Shen H., Liu Q., Garlick D., Artis D., Hensley S.E. (2014). Pneumolysin expression by *streptococcus pneumoniae* protects colonized mice from influenza virus-induced disease. Virology.

[39] Hoffmann M., Kleine-Weber H., Schroeder S., Kruger N., Herrler T., Erichsen S., Schiergens T.S., Herrler G., Wu N.H., Nitsche A. (2020). SARS-CoV-2 Cell Entry Depends on ACE2 and TMPRSS2 and Is Blocked by a Clinically Proven Protease Inhibitor. Cell.

[40] Walls A.C., Park Y.J., Tortorici M.A., Wall A., McGuire A.T., Veesler D. (2020). Structure, Function, and Antigenicity of the SARS-CoV-2 Spike Glycoprotein. Cell.

[41] McCray P.B., Pewe L., Wohlford-Lenane C., Hickey M., Manzel L., Shi L., Netland J., Jia H.P., Halabi C., Sigmund C.D. (2007). Lethal infection of K18-*hACE2* mice infected with severe acute respiratory syndrome coronavirus. J. Virol..

[42] Winkler E.S., Bailey A.L., Kafai N.M., Nair S., McCune B.T., Yu J., Fox J.M., Chen R.E., Earnest J.T., Keeler S.P. (2020). SARS-CoV-2 infection of human ACE2-transgenic mice causes severe lung inflammation and impaired function. Nat. Immunol..

[43] Oladunni F.S., Park J.G., Pino P.A., Gonzalez O., Akhter A., Allue-Guardia A., Olmo-Fontanez A., Gautam S., Garcia-Vilanova A., Ye C. (2020). Lethality of SARS-CoV-2 infection in K18 human angiotensin-converting enzyme 2 transgenic mice. Nat. Commun..

[44] Dong W., Mead H., Tian L., Park J.G., Garcia J.I., Jaramillo S., Barr T., Kollath D.S., Coyne V.K., Stone N.E. (2022). The K18-Human ACE2 Transgenic Mouse Model Recapitulates Non-severe and Severe COVID-19 in Response to an Infectious Dose of the SARS-CoV-2 Virus. J. Virol..

[45] Kumari P., Rothan H.A., Natekar J.P., Stone S., Pathak H., Strate P.G., Arora K., Brinton M.A., Kumar M. (2021). Neuroinvasion and Encephalitis Following Intranasal Inoculation of SARS-CoV-2 in K18-hACE2 Mice. Viruses.

[46] Davis M.A., Voss K., Turnbull J.B., Gustin A.T., Knoll M., Muruato A., Hsiang T.Y., Dinnon K.H., Leist S.R., Nickel K. (2022). A C57BL/6 Mouse Model of SARS-CoV-2 Infection Recapitulates Age- and Sex-Based Differences in Human COVID-19 Disease and Recovery. Vaccines.

[47] Lubkin A., Bernard-Raichon L., DuMont A.L., Valero Jimenez A.M., Putzel G.G., Gago J., Zwack E.E., Olusanya O., Boguslawski K.M., Dallari S. (2024). SARS-CoV-2 infection predisposes patients to coinfection with *Staphylococcus aureus*. mBio.

[48] Bao L., Deng W., Qi F., Lv Q., Song Z., Liu J., Gao H., Wei Q., Yu P., Xu Y. (2021). Sequential infection with H1N1 and SARS-CoV-2 aggravated COVID-19 pathogenesis in a mammalian model, and co-vaccination as an effective method of prevention of COVID-19 and influenza. Signal Transduct. Target. Ther..

[49] Kim E.H., Nguyen T.Q., Casel M.A.B., Rollon R., Kim S.M., Kim Y.I., Yu K.M., Jang S.G., Yang J., Poo H. (2022). Coinfection with SARS-CoV-2 and Influenza A Virus Increases Disease Severity and Impairs Neutralizing Antibody and CD4(+) T Cell Responses. J. Virol..

[50] Azad A.K., Rajaram M.V., Schlesinger L.S. (2014). Exploitation of the Macrophage Mannose Receptor (CD206) in Infectious Disease Diagnostics and Therapeutics. J. Cytol. Mol. Biol..

[51] Trbojevic-Akmacic I., Petrovic T., Lauc G. (2021). SARS-CoV-2 S glycoprotein binding to multiple host receptors enables cell entry and infection. Glycoconj. J..

[52] Lv J., Wang Z., Qu Y., Zhu H., Zhu Q., Tong W., Bao L., Lv Q., Cong J., Li D. (2021). Distinct uptake, amplification, and release of SARS-CoV-2 by M1 and M2 alveolar macrophages. Cell Discov..

[53] Lian Q., Zhang K., Zhang Z., Duan F., Guo L., Luo W., Mok B.W., Thakur A., Ke X., Motallebnejad P. (2022). Differential effects of macrophage subtypes on SARS-CoV-2 infection in a human pluripotent stem cell-derived model. Nat. Commun..

[54] Mezouar S., Mege J.L. (2025). Monitoring Macrophage Polarization in Infectious Disease, Lesson from SARS-CoV-2 Infection. Rev. Med. Virol..

[55] Martinez-Diz S., Marin-Benesiu F., Lopez-Torres G., Santiago O., Diaz-Cuellar J.F., Martin-Esteban S., Cortes-Valverde A.I., Arenas-Rodriguez V., Cuenca-Lopez S., Porras-Quesada P. (2022). Relevance of TMPRSS2, CD163/CD206, and CD33 in clinical severity stratification of COVID-19. Front. Immunol..

[56] Veras F.P., Pontelli M.C., Silva C.M., Toller-Kawahisa J.E., de Lima M., Nascimento D.C., Schneider A.H., Caetite D., Tavares L.A., Paiva I.M. (2020). SARS-CoV-2-triggered neutrophil extracellular traps mediate COVID-19 pathology. J. Exp. Med..

[57] Castanheira F.V.S., Kubes P. (2023). Neutrophils during SARS-CoV-2 infection: Friend or foe?. Immunol. Rev..

[58] Wang Z., Li S., Huang B. (2022). Alveolar macrophages: Achilles’ heel of SARS-CoV-2 infection. Signal Transduct. Target. Ther..

[59] Li J., Shan R., Miller H., Filatov A., Byazrova M.G., Yang L., Liu C. (2025). The roles of macrophages and monocytes in COVID-19 Severe Respiratory Syndrome. Cell Insight.

[60] Wu T.T., Travaglini K.J., Rustagi A., Xu D., Zhang Y., Andronov L., Jang S., Gillich A., Dehghannasiri R., Martinez-Colon G.J. (2024). Interstitial macrophages are a focus of viral takeover and inflammation in COVID-19 initiation in human lung. J. Exp. Med..

[61] Yeung S.T., Damani-Yokota P., Thannickal S.A., Bartnicki E., Bernier E.D., Barnett C.R., Khairallah C., Duerr R., Noval M.G., Segal L.N. (2025). Nerve- and airway-associated interstitial macrophages mitigate SARS-CoV-2 pathogenesis via type I interferon signaling. Immunity.

[62] Mitsi E., Reine J., Urban B.C., Solorzano C., Nikolaou E., Hyder-Wright A.D., Pojar S., Howard A., Hitchins L., Glynn S. (2022). *Streptococcus pneumoniae* colonization associates with impaired adaptive immune responses against SARS-CoV-2. J. Clin. Investig..

[63] Kadioglu A., Coward W., Colston M.J., Hewitt C.R., Andrew P.W. (2004). CD4-T-lymphocyte interactions with pneumolysin and pneumococci suggest a crucial protective role in the host response to pneumococcal infection. Infect. Immun..

